# Gastropods as intermediate hosts of *Angiostrongylus* spp. in the Americas: bioecological characteristics and geographical distribution

**DOI:** 10.1590/0074-02760200236

**Published:** 2020-11-27

**Authors:** Romina Valente, Maria del Rosario Robles, Julia Inés Diaz

**Affiliations:** 1 Centro de Estudios Parasitológicos y de Vectores, Buenos Aires Province, Argentina

**Keywords:** parasite, strategies of monitoring, zoonoses, intermediate host

## Abstract

**BACKGROUND:**

Intermediate hosts are key organisms in maintaining parasite life cycles, because they can act as amplifiers in the transmission from natural reservoirs to humans. One of the most important groups of intermediate hosts for zoonotic nematode infections are gastropods,slugs and snails. These are essential organisms in the larval development of *Angiostrongylus* species.

**OBJECTIVES:**

The objective of this paper is to review reports of *Angiostrongylus* spp. in naturally infected gastropods from the Americas, taking into account the diagnostic methods used in their identification, to be able to provide more accurate list of their intermediate hosts. We also discuss the factors that aid the dispersion of *Angiostrongylus* spp. in the Americas.

**METHODS:**

This study reviews scientific publications and book sections on *Angiostrongylus* spp. in the Americas, including original works assessing larvae of *Angiostrongylus* in intermediate hosts. The eligible reports were classified accordingly to their geographical location, year of first record, and the larvae identification methodologies used. Digital repositories were used for the search. The bioecological characteristics of the main intermediate hosts are summarised.

**FINDINGS:**

A total of 29 gastropod species that are naturally infected with *Angiostrongylus* spp. have been reported as intermediate hosts, 16 of which are land snails, two are freshwater snails, and 11 land slugs.

**MAIN CONCLUSIONS:**

This study highlights the importance of integrative studies, analysing both the etiological agent and its transmission dynamic in the environment, the biological and ecological characteristics of the hosts, and the impact on host populations. It is necessary to increase interdisciplinary studies to determine the potential epidemiological health risk of angiostrongyliasis in the Americas, and thus be able to establish prevention, monitoring and contingency strategies in the region.

Zoonoses are diseases transmitted from wild/domestic animals to humans.^1^ Transmission to humans is via preparation or ingestion of infected food or through close contact with infected animals.^2^ This dynamic is maintained a permanent focus of pathogen circulation in a specific geographical environment.^1^ ^,^ ^3^

Although direct contact is not frequent between humans and natural reservoirs in wildlife, exposure to a pathogenic agent can occur through any intermediate host (IH) (e.g., invertebrates, fishes) used as bait or in consumption.^4^ Intermediate hosts act as a bridge in the transmission of zoonotic pathogens, because they are amplifiers that maintain the transmission from natural reservoirs to humans. In consequence, monitoring possible IHs as pathogen transmitters is important in epidemiology, particularly for the prevention and control of emerging zoonoses.^5^

Among the invertebrates, gastropods constitute one of the most important IHs groups for zoonotic nematode infections. In the case of Angiostrongylidae (Metastrongyloidea) slugs and snails are essential organisms in which the larvae develop.^6^

Angiostrongyliasis, caused by both *Angiostrongylus cantonensis* (Chen 1935) and *Angiostrongylus costaricensis* Morera and Céspedes 1971 is a zoonotic disease. Adults of *A. cantonensis* inhabit the pulmonary artery or right ventricle of the heart, while adults of *A. costaricensis* are found in the mesenteric arteries of the caecum of their definitive host (DH) (e.g., mustelids, procyonids, felids, canids, and mainly rodents).^7^ DHs release first-stage larvae in faeces, and these utilise slugs and/or snails as IHs. The gastropods are infected by ingestion or penetration of first-stage larvae; while DHs are infected by eating infected gastropods or by contact with their slime. Transmission can also involve ingestion of paratenic hosts, such as planarias, frogs, freshwater shrimps and crabs.^8^ ^,^ ^9^ ^,^ ^10^

Species of *Angiostrongylus* have been recorded in a wide range of gastropods, indicating low host specificity for their His.^6^ ^,^ ^11^ ^,^ ^12^ In the Americas, gastropod species have been reported harbouring *Angiostrongylus* spp.,^13^ but many reports are of experimentally infected gastropods that would be unlikely to act as IHs in nature.^12^ ^,^ ^14^ In other cases, identification was performed using only morphological characteristics, but it is known that the larvae features in this genus are insufficient for identification at species level. It was therefore important to review the list of IHs and highlight the identification methods used for larvae determination.

In this study, we review reports of *Angiostrongylus* spp. in naturally infected gastropods from the Americas, taking into account the diagnostic methods used in their identification, to be able to provide more accurate list of their intermediate hosts. Also, we discuss the factors that aid the dispersion of *Angiostrongylus* spp. in the continent, mainly considering the bioecological characteristics, origin and geographical distribution of each gastropod species acting as an IH.

## MATERIALS AND METHODS

The literature reviewed in this study includes scientific publications and book sections about *Angiostrongylus* spp. in the Americas. Original papers assessing larvae of *Angiostrongylus* in IHs have been included. The reports found, up to March 2020, were in Spanish, Portuguese and English using the descriptor words: *Angiostrongylus* sp, intermediate host, angiostrongyliasis. An exhaustive search was performed using the digital repositories Scielo, Redalyc, Scopus, Dialnet, Pubmed, and Google Academic. Personal communications at congresses and conference reports were not included.

The eligible reports were classified according to their geographical location, first year of record at this site, hosts recorded at the site, and the larvae identification methodologies used. To provide a complete characterisation, previously known data on DHs compiled in Valente et al.^15^ were added to each *Angiostrongylus* species.

To understand nematode dispersion and to detect patterns, information on diet, microhabitat, environment and origin was summarised, among other bioecological characteristics of the main intermediate hosts.

## RESULTS

*Diagnosis of the larvae - Angiostrongylus* larvae were recorded in different IHs and sites throughout the Americas. Several reports of *Angiostrongylus* L3 in gastropods were based only on molecular techniques (Tables I-II). Other reports resulted from the implementation of two methods of identification, some combining morphological and experimental methods, and others using both morphological and molecular techniques. However, many records were based only on morphological characterisation of *Angiostrongylus* L3.

**TABLE I:** Geographical sites recorded, definitive hosts known, year of first larval record, intermediate host recorded and larval identification methodology, for *Angiostrongylus cantonensis* in the Americas

Geographical site	Definitive host known	first record of larvae	Intermediate host	Larval identification methodology	References
Havana, Cuba	Yes	1977	*Veronicella cubensis* (Pfeiffer 1840) *B. similaris*	Morphological-experimentation	^45^
		2015	*A. fulica*	Morphological	^53^
Puerto Rico	Yes	1984	*S. octona*	Morphological-experimentation	^46^
Santo Domingo, Dominican Republic	Yes	1991	*S. octona*	Morphological	^54^
Jamaica	Yes	2000	*Thelidomus aspera* (Férrusac 1821)	Morphological	^55^
		2013	*Pleurodonte* sp. *Sagda* sp. *Poteria* sp.	Morphological	^56^
Espírito Santo, Brazil	Yes	2007	*S. octona* *A. fulica* *B. similaris*	Molecular PCR-RFLP (ITS2/ClaI enzyme)	^57^
			
		2010	*S. linguaeformis*	Molecular PCR-RFLP (ITS2/ClaI enzyme)	^13^
São Paulo, Brazil	Yes	2007	*A. fulica* *S. octona*	Molecular PCR-RFLP (ITS2/ClaI enzyme)	^57^
		2009	*S. linguaeformis*		^13^
		2011	*B. similaris*		
Paraná, Brazil	No	2008	*A. fulica*	Molecular PCR-RFLP (ITS2/ClaI enzyme)	^13^
Bahia, Brazil	No	2009	*A. fulica* *B. similaris* *S. octona* *S. linguaeformis*	Molecular PCR-RFLP (ITS2/ClaI enzyme)	^13^
Pernambuco, Brazil	No	2008	*A. fulica* *P. lineata*	Morphological-molecular PCR- RFLP(ITS2/ClaI enzyme)	^58^
Pará, Brazil	Yes	2009	*A. fulica* *S. octona*	Molecular PCR (COI), PCR-RFLP(ITS2/ClaI enzyme)	^59^
		2010	*S. linguaeformis*		^13^
Santa Catarina, Brazil	No	2009	*B. similaris*	Molecular PCR-RFLP (ITS2/ClaI enzyme)	^13^
		2010	*A. fulica*		
Rio de Janeiro, Brazil	Yes	2010	*A. fulica*	Morphological-experimentation	^47^
Amazonas, Brazil	No	2014	*A. fulica*	Morphological	^60^
Sergipe, Brazil	No	2016	*A. fulica* *Cyclodontina fasciata* (Potiez & Michaud 1838) *Bulimulus tenuissimus* (d’Orbigny 1835)	Molecular PCR (COI)	^61^
Guayas, Ecuador	Yes	2008	*A. fulica* *P. lineata*	Morphological-experimentation	^41^
Louisiana, USA	Yes	2013	*P. maculata*	Molecular PCR(18S)	^62^
Florida, USA	Yes	2013	*A. fulica*	Molecular qPCR (ITS1)	^63^
		2015	*Alcadia striata* (Lamarck 1822) *B. similaris* *Zachrysia provisoria* (Pfeiffer 1858)	Molecular qPCR (ITS1)	^51^
		2017	*Paropeas achatinaceum* (Pfeiffer 1846) *Succinea floridana* Pilsbry 1905 *Ventridens demissus* (Binney 1843) *Zonitoides arboreus* (Say 1816)	Molecular qPCR (ITS1)	^43^
Colombia	No	2014	*A. fulica*	Morphological	^64^

PCR-RFLP: polymerase chain reaction-restriction fragment length polymorphism; qPCR: quantitative polymerase chain reaction.

**TABLE II:** Geographical sites recorded, definitive hosts known, year of first larval record, intermediate host recorded and larval identification methodology, for *Angiostrongylus costaricensis* in the Americas

Geographical site	Definitive host known	First record of larvae	Intermediate host	Larval identification methodology	References
Costa Rica	Yes	1970	*S. plebeia* *Diplosolenodes occidentalis*	Morphological	^65^
Paraná, Brazil	Yes	1991	*L. maximus* *B. similaris* *L. flavus*	Morphological	^32^
Rio Grande do Sul, Brazil	Yes	1993	*Cornu aspersum* *P. variegatus* *P. soleiformis* *B. angustipes* *B. similaris*	Morphological	^29^ ^,^ ^66^
	2018	*Meghimatium pictum*	Morphological-experimentation	^67^
Santa Catarina, Brazil	Yes	1999	*P. variegatus* *S. linguaeformis* *D. laeve*	Morphological	^68^
Ecuador	Yes	1993	*S. plebeia*	Morphological	^66^
Honduras	Yes	1993	*S. plebeia*	Morphological	^66^
Nicaragua	Yes	1993	*S. plebeia*	Morphological	^66^

Most reports of *A. cantonensis* were based on experimental and/or molecular methods, while most of the records of the other *Angiostrongylus* spp. larvae have only been based on morphological analyses.

In addition, on several occasions the susceptibility to infection of different gastropod species has been evaluated with positive results, as in the case of *Biomphalaria* spp. and *Pomacea canaliculata* (Lamarck 1822).^12^ ^,^ ^14^ ^,^ ^16^ However, this study includes a list only of naturally infected IHs.

*Intermediate hosts and their bioecological characteristics -* Table III shows the bioecological characteristics of the 29 gastropod species reported as IHs of *Angiostrongylus* spp. and the ecological and environmental features associated with these.

**TABLE III:** American distribution and bioecological characteristics of naturally intermediate hosts reported for *Angiostrongylus* spp.

Host species	Distribution	Diet	Microhabitat	Environment	Origin*	Human consumption	Sex	Estivation hibernation	Activity	Reprod. rate **	Parasite
*Achatina fulica*	Original of East África. In the Americas: Argentina, Bolivia, Brazil, Caribbean Islands, Colombia, Cuba, Dominican Republic, Ecuador, Paraguay, Peru, Trinidad and Tobago, USA,Venezuela	Polip Copro	Domiciliary pest	Trop/Subtrop	Exotic	Yes	Her	Yes	Noct	Hight	Aca
*Bradybaena similaris*	Original of East Asia. In the Americas: Argentina, Brazil, Colombia, Cuba, Paraguay, Puerto Rico, Uruguay, USA	Polyp	Domiciliary pest	Trop/Subtrop	Exotic	No	Her	Yes	Noct	Low	Aca Aco
*Subulina octona*	Original of Europe. Caribbean Sea. In South America: Argentina, Brazil, Colombia, Ecuador	Herb	Domiciliary pest	Trop/Subtrop	Exotic	No	Her	No	Diur	Low	Aca
*Veronicella cubensis*	Cuba, Dominican Republic, Puerto Rico, Lesser Antilles	Herb	Domiciliary pest	Trop/Subtrop	Native	No	Her	No	Noct	Low	Aca
*Thelidomus aspera*	Jamaica	Herb	Domiciliary pest	Trop/Subtrop	Native	No	Her	Unknown	Diur	Low	Aca
*Pleudodonte* sp.	Jamaica	Herb	Domiciliary	Trop/Subtrop	Native	No	Her	Unknown	Diur	Low	Aca
*Sagda* sp	Jamaica	Herb	Domiciliary	Trop/Subtrop	Native	No	Her	Unknown	Diur	Low	Aca
*Poteria* sp*.*	Jamaica	Herb	Domiciliary	Trop/Subtrop	Native	No	Her	Unknown	Diur	Low	Aca
*Pomacea lineata*	Brazil, Guyana, French Guyana, Surinam	Detr	Aquatic	Trop/Subtrop	Native	No	Dio	Yes	Diur	Low	Aca
*Pomacea maculata*	Argentina, Bolivia, Brazil, Ecuador, Paraguay, Peru, Uruguay	Detr	Aquatic	Trop/Subtrop	Native	No	Dio	Yes	Diur	Medium	Aca
*Cyclodontina fasciata*	Brazil	Herb	-	Trop	Native	No	Her	Unknown	Noct	Low	Aca
*Bulimulus tenuissimus*	Brazil and USA	Herb	Domiciliary	Trop/Subtrop	Native	No	Her	No	Noct	Low	Aca
*Zachrysia provisoria*	Cuba and USA	Herb	Domiciliary pest	Trop/Subtrop	Native	No	Her	Unknown	Noct	Low	Aca
*Alcadia striata*	Caribbean Islands and USA	Herb	Domiciliary pest	Trop/Subtrop	Native	No	Her	Unknown	Noct	Low	Aca
*Paropeas achatinaceum*	Original of Southeast Asia. Currently in USA	Herb	Domiciliary	Trop/Subtrop	Exotic	No	Her	No	Diur	Low	Aca
*Succinea floridana*	USA	Herb	Damp habitat	Trop/Subtrop	Native	No	Her	No	Diur	Low	Aca
*Ventridens demissus*	USA	Herb	Domiciliary	Trop/Subtrop	Native	No	Her	No	Diur	Low	Aca
*Zonitoides arboreus*	USA, Central America, Argentina	Herb	Domiciliary	Trop/Subtrop	Native	No	Her	No	Diur	Low	Aca
*Cornu aspersum*	Original of Europe. Currently in Canada, USA, Mexico, Dominic Republic, Puero Rico, Guatemala, Costa Rica Argentina, Brazil, Chile, Colombia, Ecuador, Guyana, Peru, Uruguay, Venezuela	Herb	Domiciliary	Trop/Subtrop	Exotic	Yes	Her		Noct	Low	Aco
*Sarasinula plebeia*	Original of New Caledonia. Currently in USA, Mexico, Caribbean Islands, Central America, Brazil, Colombia, Ecuador, Peru,Venezuela	Herb	Domiciliary pest	Pantrop	Exotic	No	Her	Yes	Noct	Low	Aco
*Sarasinula linguaeformis*	Argentina, Brazil, Colombia, Dominica, Ecuador, Guadalupe, Guyana, Peru	Herb	Domiciliary pest	Trop/Subtrop	Native	No	Her	Yes	Noct	Low	Aco
*Limax maximus*	Original of Europe. Currently in Canada, USA, Mexico, El Salvador, Honduras, Hawaii, Argentina, Brazil, Chile, Colombia	Herb	Domiciliary	Template	Exotic	No	Her	No	Noct	Low	Aco
*Limacus flavus*	Original of Europe and Asia. Currenty in Argentina, Brazil, Canada, Colombia, Ecuador, Mexico, Uruguay, USA	Herb	Domiciliary	Template	Exotic	No	He.	No	Noct	Low	Aco
*Phyllocaulis variegatus*	Argentina, Brazil, Paraguay, Uruguay	Herb	Domiciliary pest	Trop/Subtrop	Native	No	Her	Yes	Noct	-	Aco
*Phyllocaulis soleiformis*	Argentina, Bolivia, Brazil, Uruguay	Herb	Domiciliary pest	Trop/Subtrop	Exotic	No	Her	Yes	Noct		Aco
*Deroceras laeve*	Original of Europe. Currently in Canada, USA, Mexico, Jamaica, Costa Rica, Hawaii, Dominica, Argentina, Brazil, Chile, Colombia, Peru, Paraguay, Uruguay	Herb	Domiciliary	Template	Exotic	No	Her	No	Noct		Aco
*Belocaulus angustipes*	USA, Honduras, Argentina, Brazil	Herb	Domiciliary pest	Trop/Subtrop	Native	No	Her	Yes	Noct		Aco
*Diplosolenodes occidentalis*	Central America, Jamaica, Lesser Antilles, Colombia, Ecuador, Venezuela	Herb	Domiciliary pest	Trop/Subtrop	Native	No	Her	Yes	Noct		Acos
*Meghimatium pictum*	Original of Eastern and Southern Asia. In South America: Argentina, Brazil	Heb	Domiciliary pest	Trop/Subtrop	Exotic	No	Her	No	Noct		Aco

Trop/Subtrop: tropical/subtropical; Pantrop: pantropical; Reprod. rate in comp. with *A. fulica*: reproduction rate in comparison with *A. fulica*; Polyp: polyphagous; Copr: coprophagous; Herb: herbivorous; Detr: detritivorous; Her: hermaphrodite; Dio: dioco; Noct: nocturnal; Diur: diurnal; Aca: *Angiostrongylus cantonensis*; Aco: *Angiostrongylus costaricensis*. *: the terms native refers to its origin in the Americas; **: reprod. rate in comp. with *A. fulica*: high: > 500 eggs; medium: > 250 eggs ≤ 500 eggs; low: ≤ 250 eggs.

*Bradybaena similaris* (Férrusac 1822), *Achatina fulica* Bowdich 1822, *Sarasinula linguaeformis* (Semper 1885) and *Subulina octona* (Bruguiére 1789) are the most frequent land gastropods reported as IHs of *A. cantonensis* in the Americas. Some of these species are considered crop pests due to their trophic habits and *A. fulica* is known to also have coprophagic habits.^17^ These gastropods are found in residential areas, constituting a link between the synanthropic definitive hosts and humans, indicating a beneficial scenario for the development of *A. cantonensis*.^17^ ^,^ ^31^

Among the IHs, *A. fulica* has a wide geographical distribution in the Americas (see Table III).^9^ ^,^ ^18^ ^,^ ^19^ ^,^ ^20^ ^,^ ^21^ ^,^ ^22^ ^,^ ^23^ This invasive gastropod has high reproductive rates and periods of estivation/hibernation, and as the most invasive species, its particular biological characteristics give it adaptive plasticity, and favour its role as the main IH for *A. cantonensis*.^5^ ^,^ ^24^ Among freshwater IHs of *A. cantonensis* (Table III), only *Pomacea lineata* (Spix 1827) and *Pomacea maculata* (= *P. insularum*) Perry 1810 were reported.^25^ ^,^ ^26^ ^,^ ^27^ The lack of records of *Pomacea canaliculata* (Lamarck 1822) as an IH is striking, because its distribution in Brazil overlaps that of *P. maculata*. *P. canaliculata* is found in Argentina, Brazil, Chile, Colombia, Ecuador, Paraguay, Uruguay,^25^ ^,^ ^27^ and is the main freshwater IH of *A. cantonensis* in Asia.^28^

In the case of *A. costaricensis*, the reports of naturally infected IHs are mainly associated with land slugs: *Belocaulus angustipes* (Heynemann 1885), *Deroceras laeve* (Müller 1774), *Diplosolenodes occidentalis* (Guilding 1825), *Limacus flavus* (Linnaeus 1758), *Limax maximus* (Linnaeus 1758), *Meghimatium pictum* (Stoliczka 1873), *Sarasinula linguaeformis* (Semper 1885), *Sarasinula plebeia* (Fischer, 1868), *Phyllocaulis soleiformis* (d’Orbigny 1835) and *Phyllocaulis variegatus* (Semper 1885), with the only land snail reported being *Cornu asperum* (Müller 1774) (Thiengo et al.^29^). These gastropods are considered as pests due their herbivorous habits. During estivation periods, some of them produce mucus around their bodies that isolate them from unfavourable conditions.^30^ ^,^ ^31^

Several reports show a wide variety of IHs experimentally infected with *A. costaricensis* such as, *B. glabrata* (Say 1818), *B. straminea* (Dunker 1848), *B. tenagophila* (d’Orbigny 1835), *Phyllocaulis boraceiensis* Thomé 1972 and *Megalobulimus abbreviatus* (Bequaert 1948), and all these gastropod species are susceptible to develop infective larvae.^14^ ^,^ ^32^ ^,^ ^33^

For the remaining *Angiostrongylus* species only two gastropods, *B. glabrata* and *Polygra septemvolva* (Say 1818), have been reported as intermediate hosts of *A. schmidti* Kinsella 1971,^11^ and *B.glabrata* and *P. canalicuta* as IHs of *A. vasorum* (Bailliet 1866).^12^ ^,^ ^16^ ^,^ ^34^ However, all these reports come from experimental gastropod infestations.

Most studies have focused on the gastropod species that are already known as IHs for *Angiostrongylus*. Therefore, it is possible that the number of IH species in the Americas has been underestimated.

*Geographical distribution - Angiostrongylus* larvae were recorded in 11 American countries, with Brazil being the country where most studies were performed. *A. cantonensis* is widely distributed in Central and South America (Table I), but there is no evidence that it was introduced from the western hemisphere by its DH (e.g., *Rattus* spp.) or its IH (e.g., the invasive gastropod *A. fulica*).^4^ ^,^ ^35^ ^,^ ^36^ In contrast, *A. costaricensis* is not widely spread in the Americas, even though it is an endemic species (Table II).

The literature shows that *Angiostrongylus felineus* Vieira et al. 2013, *A. gubernaculatus* Dougherty 1946, *A. lenzii* Souza, Simões, Thiengo et al. 2009, *A. morerai* Robles, Navone and Kinsella 2008, *A. raillieti* Travassos 1927 and *A. schmidti* have a limited and overlapping geographical distribution in the Americas.^10^ ^,^ ^15^ Their DHs are wild animals, and there are no reports of these *Angiostrongylus* species in synanthropic hosts from urban/agricultural areas. Furthermore, there are no records of their IHs, except for *A. vasorum* and *A. schmidti* from experimental research (Table II). Their zoonotic potential cannot be evaluated due to the lack of reports of their definitive and intermediate hosts.^10^

*Native and exotic gastropods -* Exotic species can influence ecosystem health by introducing parasites from their native area, or by amplifying parasites already existing in the introduced range.^37^ Some invasive species can impact human health by carrying zoonotic parasites. When an exotic parasite reaches a native host species, the phenomenon is called “spillover”. When a native parasite infects an invasive host, leading to increased opportunities to infect autochthonous species, the phenomenon is called “spillback”.^37^ One of the causes that favours these interactions is the low host specificity of the parasite.^38^

Examples of low host specificity in this work are *A. cantonensis* and *A. costaricensis*, since both exotic and native species are involved as IHs. In the first case, only four of the 18 IH species reported are exotic: *A. fulica*, *B. similaris*, *Subulina octona* and *Paropeas achatinaceum* (Pfeiffer 1846) (Table III). *Achatina fulica* is native to Africa, being observed in the Americas for the first time in Hawaii in 1936, and later it was reported in the rest of the continent, except Chile and Uruguay.^39^ ^,^ ^40^ ^,^ ^41^ *Bradybaena similaris* is native to Asia, being observed for first time in the Americas in Brazil in 1835, and later it spread to almost all South America (Table III).^23^ ^,^ ^42^ *S. octona* originated in Europe, being recorded in South America in 1914,^23^ while whereas *P. achatinaceum* is native to Southeast Asia.^43^

*Angiostrongylus costaricensis* is endemic to the Americas and almost the same number of exotic and of endemic gastropod species have been reported as IHs (6 *vs* 5) (Table III).

Thus, the dispersion of *Angiostrongylus* spp. in the Americas is favoured by low host specificity and the high number of naturally infected gastropod species.

## DISCUSSION

The present study lists all the records and IHs identified as reservoirs of *Angiostrongylus* spp. larvae in the Americas. Reports were analysed, taking into account the diagnostic methods. Additionally, the bioecological characteristics, origin and geographical distribution of the IHs recorded in the literature were also summarised.

Currently, only two of the nine *Angiostrongylus* species distributed in the Americas have been confirmed as zoonotic agents (i.e., *A. cantonensis* and *A. costaricensis*). Nevertheless, the potential health risk of the rest of the *Angiostrongylus* species remains unknown.

Identification of larvae of *Angiostrongylus* spp. by morphological methods is very difficult. The main morphological characteristics used by the authors are shape and body length, oesophagus length, excretory pore to the anterior end distance, genital primordium to posterior end distance, tail length, and tip of tail.^44^ Unfortunately, the small size (400-600 µm) and the absence of diagnostic morphological characters developed of the infective larvae (L3) does not allow a good identification.

Fortunately, in recent years the diagnostics of parasites has been supported by experimental and molecular tools.^45^ ^,^ ^46^ ^,^ ^47^ There are two types of experimental methods: in one case, captive mammals (e.g., *Rattus* sp.) are fed with a pool of live larvae (L3) obtained from naturally infected IH (e.g., *A. fulica*). After 28 days post infection, immature (L4-L5) and adult worms are found in the DH, of which accurate morphological identification is possible (e.g., Andersen et al.^46^). In other cases, first-stage larvae are isolated from the faeces of the DH. Gastropods are fed with a pool of isolated larvae (L1). After 30 days post infection, L3 that emerge from the IH are identified morphologically.^11^ ^,^ ^12^ ^,^ ^16^

In the case of the molecular methods, gastropods are artificially digested according to the Wallace & Rosen^48^ and Baermann-Moraes techniques.^15^ After sedimentation, the material is analysed under a stereomicroscope and the larvae recovered from each collection pool are identified following DNA extraction and sequencing methods.

From 1977 to 2007, all the reports of *A. cantonensis* larvae were based on morphological and experimental methods, and they were recorded only in Central America. From 2007 to the present, its larvae were identified by molecular techniques, and were focused in South America. In contrast, all identifications of *A. costaricensis* larvae were carried out only by morphological methods. From 1999 until now, no new gastropod species have been added as IHs for *Angiostrongylus* spp., but probably the range of IHs has been underestimated. In the cases of *A. vasorum* and *A. schmidti* in the Americas, the larvae were obtained by experimental methods, presenting a similar eco-epidemiological scenario to that of *A. costaricensis.*

Host-parasite interactions are affected by genetic, biological and ecological aspects of both members of the relationship. Land gastropod species, such as *A. fulica*, *B. similaris*, *Phyllocaulis* spp., *Sarasinula* spp. and *Belocaulus angustipes*, show some bioecological characteristics that would favour *Angiostrongylus* spp. dispersion, such as polyphagia, peridomiciliary restriction, nocturnal habits, and estivation/ hibernation periods.^17^ These IHs act as a link between synanthropic definitive hosts and humans. Additionally, unlike other species of gastropods, *A. fulica* is eaten raw by humans in many regions (e.g., Ecuador, Jamaica), increasing the risk of disease.^41^

Environment plays an important role in the interaction between *Angiostrongylus* spp. and their IHs. Any disturbances in the structure and function of the ecosystem could indirectly influence zoonoses transmission.^49^ The conversion of natural habitats to agricultural land, pastures and plantations due to the expanding human population has caused the displacement of wildlife and the wild/urban interface, causing more contact between humans and wild animals.^5^ ^,^ ^50^ In this context, it is very important to continuously monitor different environments, to detect those changes that could favour the installation of a new focus of infection.

Introduction of exotic species could affect the equilibrium of an ecosystem by the invasion of new parasite species in native hosts, or amplify the parasites that already exist in the area.^37^ The former seems to be the case in the introduction of *A. cantonensis* in the Americas, which has been able to invade at least 14 native gastropod species by “spillover”. On the other hand, *A. costaricensis*, which parasitises native species, was able to invade exotic gastropods, also favouring its dispersion by “spillback”. Apparently, both phenomena added to the low host specificity that these *Angiostrongylus* species seem to have, favouring the dispersion of these zoonoses in the Americas. The current paper demonstrates that the spread of *A. cantonensis* and *A. costaricensis* through gastropod IHs in the Americas is not limited to exotic or native origins. As has been stated, the success in obtaining larvae of *Angiostrongylus* spp. by infecting different freshwater gastropods denotes their low host specificity,^43^ ^,^ ^51^ which constitutes a risk since it favours the dispersion of the species.

The information in the present review about the IHs of *Angiostrongylus* spp. complements the previous study of Valente et al.^15^ on DHs, and sumarises all the available information in the Americas. In addition, this study highlights the importance of integrative studies, analysing both the etiological agent and the dynamic of its transmission in the environment, the biological and ecological characteristics of its hosts, and the impact on host populations. This is in agreement with the concept of “One Health” that states that human and animal health are interdependent and are linked to the environment in which they coexist. In this way, the etiological agent, the host and the environment constitute a triad that is influenced by continuous variations caused by environmental and social changes.^52^

It is necessary incrase interdisciplinary studies to determine the potential epidemiological health risk of angiostrogiliasis in the Americas, and thus be able to establish prevention, monitoring and contingency strategies in the region.
